# Berberine improves insulin-induced diabetic retinopathy through exclusively suppressing Akt/mTOR-mediated HIF-1α/VEGF activation in retina endothelial cells

**DOI:** 10.7150/ijbs.62868

**Published:** 2021-10-20

**Authors:** Ning Wang, Cheng Zhang, Yu Xu, Hor-Yue Tan, Haiyong Chen, Yibin Feng

**Affiliations:** 1 School of Chinese Medicine, The University of Hong Kong; 2 Centre for Chinese Herbal Medicine Drug Development, School of Chinese Medicine, Hong Kong Baptist University

**Keywords:** Berberine, Insulin, Diabetic Retinopathy, VEGF, HIF-1α, Akt/mTOR

## Abstract

**Background:** Insulin therapy is the major treatment of glycaemic control in type I diabetes mellitus (DM) and advanced type II DM patients who fail to respond to oral hypoglycemic agents. Nonetheless, insulin therapy is deemed unsuccessful in controlling the incidence of diabetic retinopathy (DR) and is likely a risk factor. Berberine, an isoquinoline alkaloid, has caught great attention towards its anti-diabetic mechanisms. This study aims to investigate the effect of berberine in decelerating DR progression in insulin-treated DM.

**Methods:** To better understand the therapeutic potential of berberine in the presence of insulin, we elaborated the action of mechanism whether berberine inhibited retinal expression of HIF-1α and VEGF through regulating AKT/mTOR pathway. Suppression of insulin-induced neovasculature of retina endothelial cells by berberine was also studied. Lastly, the *in vivo* efficacy and safety of berberine as adjuvant therapy for the treatment of DR were systemically investigated in experimental type I and type II DM mice with insulin treatment.

**Results:** Among various types of retinal cells, the activity of HIF-1α and VEGF in retinal endothelial cells could be particularly and exclusively stimulated by insulin intervention, which could be inhibited by berberine treatment in a dose- and time-dependent manner. Berberine suppressed Akt/mTOR activity in these cells, and restoration of Akt/mTOR signalling attenuated berberine's inhibition on HIF-1α and VEGF expression. Berberine suppressed the progression of DR in experimental type I and type II diabetic mice receiving insulin therapy.

**Conclusion:** Berberine improves insulin-induced diabetic retinopathy in type I and II diabetes through inhibiting insulin-induced activation of retinal endotheliocytes via Akt/mTOR/ HIF-1α/VEGF pathway.

## Introduction

Diabetic retinopathy (DR) is the major cause of vision loss in patients with Diabetes Mellitus with a characteristic of microvascular dysregulation 1 and progresses from non-proliferative to proliferative stage upon loss of proper glycaemic control 2. It has been believed that the vascular endothelial growth factor (VEGF) plays a dominant role during the initiation and progression of DR, whose transcriptional expression in the retina was controlled by hypoxia-inducible factor-1α (HIF-1α) 3. Retina VEGF induces the neovasculature, as well as leakage of neighbouring capillaries, and in subsequence destroy the retina structure and therefore cloud the vision.^4^. Currently, anti-VEGF treatment has been approved for the late-stage, proliferative DR to reduce the formation the new blood vessels, following the panretinal photocoagulation and vitrectomy as the primary DR treatment 5. Monoclonal antibody against VEGF has shown its effectiveness in the clinical trials, however, its application was somehow limited due to the high cost of the treatment or unsatisfactory response rate in some patients 2.

Proper glycaemia control in diabetic patients shows improvement in DR incidence 6. Insulin therapy is the important mean of glycaemic control of diabetic patients. Biosynthetic or animal-source insulin is the most effective way for the management of type I diabetic patients as well as in patients with advanced type II diabetes who fail to well respond to oral hypoglycemic agents. However, insulin therapy is deemed to be unsuccessful in controlling the incidence of DR and likely to be one of its risk factors^7^. Diabetic patients in the United States who received insulin treatment experienced risk of DR progression unexpectedly 8. Compared with those without insulin treatment, type II diabetic patients using insulin got a higher possibility of DR progression (39% in the non-insulin treatment group versus 70% in the insulin treatment group) 9. In Latinos with familial type II diabetes, studies showed that the DR prevalence and severity were associated with their residual endogenous insulin secretion 10. A recent meta-analysis collecting data from seven cohort studies showed a significant association between the risk of DR with insulin intervention, despite that, they also detected existing heterogeneity of the literature 11. Experimentally, it is reported that insulin induces retinal neovascularization via inducible expressions of HIF-1α and VEGF in human retinal endothelial cells, which forms new blood vessels during DR progression 12. Insulin activates the phosphatidylinositol 3-kinase/target of rapamycin (AKT/mTOR) pathway to induce HIF-1α and VEGF 13. Moreover, rapid accumulation of insulin via exogenous administration triggers VEGF expression 14.

Berberine (BBR) is a natural bioactive alkaloid derived from a variety of Chinese Medicinal herbs including *Rhizoma Coptidis*. Synthetic production of BBR allows the compound to be widely available as an affordable over-the-counter (OTC) drug in China for years. It has been reported with various pharmacological effects such as anti-inflammation, anti-oxidation, hepatic protection and anti-cancer 15-17. The hypoglycemic activity of BBR was intensively reported by a series of experimental studies, nonetheless, systematic review and meta-analysis showed the incomparable glycaemia control of BBR than other oral hypoglycemic drugs 18. Recent studies showed BBR protected retinal endothelial cells from leukocytes attack, which further reduced capillary degeneration in diabetic patients 19. Although the clinical indication has yet to be concluded, experimental evidence on deteriorating DR condition in insulin-treated Diabetic Mellitus has been observed in diabetic mice 20. It remains unknown whether BBR can improve DR in type I and type II diabetes with insulin therapy.

In this study, we systematically investigated the anti-DR effect of BBR in insulin-treated diabetes. The effects of insulin on inducing VEGF expression were examined in different specific types of retina cells and the time- and dose-dependent inhibitory effects of BBR on HIF-1α and VEGF were determined. The activities of proliferation, migration and increased membrane permeability of the retinal endothelial cells that were critically involved in the retina neovascularization, in the presence of BBR, was measured. The efficacy and safety of BBR with insulin in improving DR were elaborated in experimental in type I and type II diabetes mellitus mice receiving insulin treatment. In this study, we systematically investigated the anti-DR properties of BBR in insulin-treated diabetes.

## Materials and Methods

### Chemicals, antibodies, plasmids and reagents

Streptozotocin (STZ), berberine chloride and insulin were purchased from Sigma-Aldrich (USA); Antibodies against HIF-1α, phosphor-Akt, Akt, phosphor-mTOR, mTOR and β-actin were purchased from Abcam (UK) or Cell Signaling Technologies (USA); Constantly activated plasmid of Akt and mTOR, pFBD-Akt1(T308A/S473A, #86578) and pcDNA3-FLAG-MTOR-R2505P (#69015) respectively, were obtained from Addgene (USA); 21, 22 ELISA kits for VEGF detection were purchased from Excell Bio (China).

### Cells and cell culture

Retinal pigmented epithelium cell line ARPE-19, Retina Müller cell line rMC-1, astrocyte cell line fetal-hTERT, vascular endothelial cell line HREC and ganglion cell line RGC-5 were cultured with appropriate culture medium supplemented with 10% Fetal bovine serum (FBS) and 1% penicillin/streptomycin at 37℃, 5% CO_2_.

### Cell assays

The proliferation of endothelial cells was determined by MTT assay; migration of endothelial cells was examined by transwell assay; The permeability of endothelial cells was quantified by dextran-FITC penetration through endothelial monolayer. All the assays have been established in our previous publication 23.

### Animal study

Experimental procedures of animal study have been endorsed by the Committee on the Use of Live Animals and Teaching and Research (CULATR) of the University of Hong Kong.

*Animal models* Both experimental type I and II diabetic mice models were used. For the study of type I diabetes mellitus, we used streptozotocin (STZ)-induced hyperglycemic mice. 10-week C57/BL/J mice received 5 constitutive intraperitoneal injections of 50 mg/kg STZ in citric buffer (pH4.5). Mice with 4h-fasting blood glucose (FBG) within 16.0 to 25.0 nmol/L were qualified to randomisation. Mice only injecting citric buffer were used as normal control. For the study of type II diabetes mellitus, we used mice homozygous for the diabetes spontaneous mutation (C57BLKS-Lepr*^db^*) as a typical type II diabetic model. C57BLKS-Lepr*^db^* mice spontaneously develop obesity, insulin resistance and elevation of blood sugar around 4 to 8 weeks after birth. 10-week C57BLKS-Lepr*^db^* mice with FBG within 16.0 to 25.0 nmol/L were included in the study.

*Treatment regimens* In both type I and type II diabetic model, recombinant insulin was given in accordance with the published standard protocol 24. Mice received a single injection of 0.6 U of Lantus (insulin glargine, recombinant DNA origin) every day. BBR was delivered by oral and ocular administration. Doses of oral administration (50 and 100 mg/kg) ocular delivery (0.2 and 0.4 μg/kg) were applied; Treatment lasted for 12 weeks. Mice in the normal group received a vehicle as control.

### Fundus Photography

Fundus photography is adequate for measuring the severity of retinopathy, including changes of vessel calibre, swelling of vessels, abnormal new growth of vessels on retinal surface and tortuosity. Fundus photography was performed adopted from published protocol 25, 26.

### Measurement of retinal vascular leakage

*In vivo* measurement of retinal vascular leakage was conducted with fluorescein angiography with a protocol adapted from previous publications 27-29. Images were captured under a confocal laser microscope (200×, Carl Zeiss LSM 780, USA).

Evans blue assay was used to quantitatively measure the retina vascular leakage. Evans blue was injected into the mice intravenously and retina Evan blue dye leakage was measured accordingly to our previous publication 2.

### Measurement of retinal neovascularization

Retinal vascular preparation assay was applied as a direct indicator of retinal neovascularization. Freshly fixed eyeballs were digested with 3% trypsin dissolved in 0.1M Tris buffer (pH=8.2), followed by hematoxylin-eosin staining. The retina vasculature has been then imaged the microscope (LEICA) with 400× magnification. The number of endothelial cells/ pericytes and acellular capillaries were quantified in each scope. Each sample was investigated by two individual researchers. Total and laminar retinal thicknesses were measured as a secondary indicator of proliferative DR.

### Safety measurement

At the end of the animal study, the liver (AST/ALT) and kidney function (BUN/Creatinine) were measured. Blood tests and histological measurements of lungs and heart were performed.

### RNA extraction and quantitative real-time PCR

Total RNA extraction was performed by the Trizol method (Takara, Japan). cDNA was prepared from total RNA with a first-strand synthesis kit (Takara, Japan). Expression of targeted genes was determined by quantitative real-time PCR using SYBR Green (Takara, Japan) on the LC480 platform (Roche, USA). The expression level of β-actin was used as the internal control. Primer sequence would be available upon request.

### Immunoblotting

Protein from cell or tissue samples was extracted using RIPA buffer and loaded onto the SDS-PAGE for separation by electrophoresis. Proteins were then transferred to the PVDF membrane. Followed by blocking by 5% BSA in TBST buffer (25 mM Tris-HCl, 137 mM NaCl and 2.7 mM KCl, 0.05% Tween-20, pH 7.4 ± 0.2) for 2 hours at room temperature, the membranes were incubated with the appropriate primary antibodies of interest overnight at 4 ^0^C and secondary antibodies for 2 hours at room temperature. The membrane was read with ECL select substrate (GE Healthcare, Germany) on the Chemidoc chemiluminescent platform (Biorad, USA).

### Statistical Analysis

Data was present in mean ± SD. An ordinary two-way ANOVA was used to measure the difference of multiple groups, while a student t-test was applied for the measurement of difference between paired group co. p < 0.05 were considered as statistically significant.

## Results

### Insulin-induced expression of VEGF and HIF-1α specifically in retina endothelial cells

**Figure 1:**
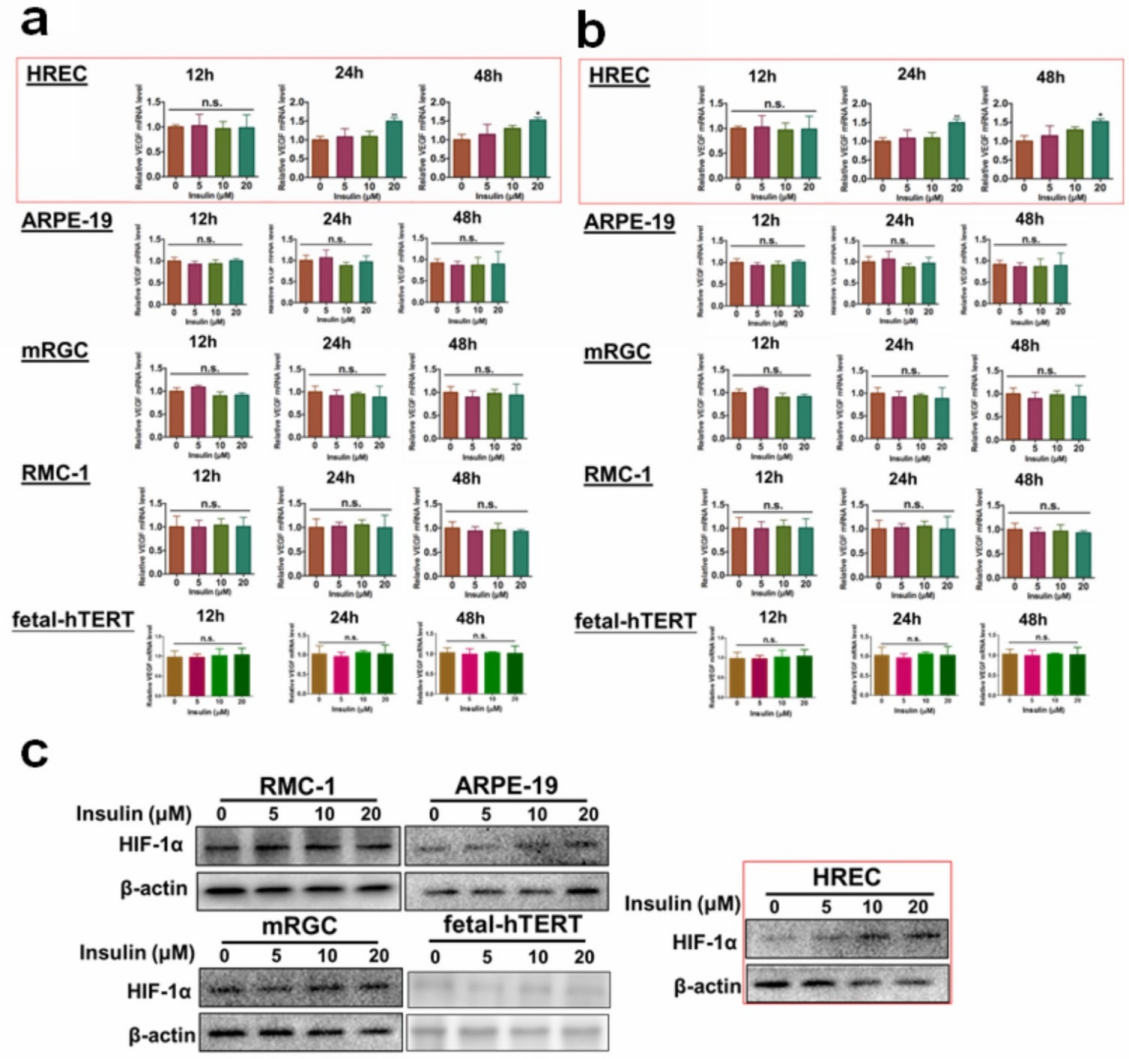
** Insulin-induced VEGF and HIF-1α expression particularly in retina endothelial cells.** Insulin induces mRNA expression **(a)** and protein expression** (b)** of VEGF in HRECs but not other cell types. **c.** insulin induces HIF-1α expression in HRECs but not other cell types.

Several different types of cells can be found in the retina. To understand which cell type could respond to insulin treatment in terms of VEGF production, we, first of all, examined the mRNA and protein expression of VEGF in insulin-treated retinal pigmented epithelium cell line ARPE-19, Retina Müller cell line rMC-1, astrocyte cell line fetal-hTERT, vascular endothelial cell line HREC and ganglion cell line RGC-5. Different doses of insulin (0, 5, 10, 20 μM) were tested, and the expression of VEGF was examined after different time of insulin was given (12,24,48 hours). It was shown that only HREC cells could significantly respond to insulin treatment at a dose above 10 μM for at least 24 hours (Fig. 1a-b). As hypoxia-inducible factor-1α (HIF-1α) is the responsible transcriptional factor of VEGF production, we then tested if insulin treatment induced HIF-1α expression in retina cells. Consistently, we observed that only HREC cells treated with insulin exhibited an increase in HIF-1α expression (Fig. 1c). Taken together, these observations concluded that endothelial cells were the major responsive cell type in the retina during insulin treatment.

### BBR significantly suppressed expression of VEGF and HIF-1α in retina endothelial cells

**Figure 2:**
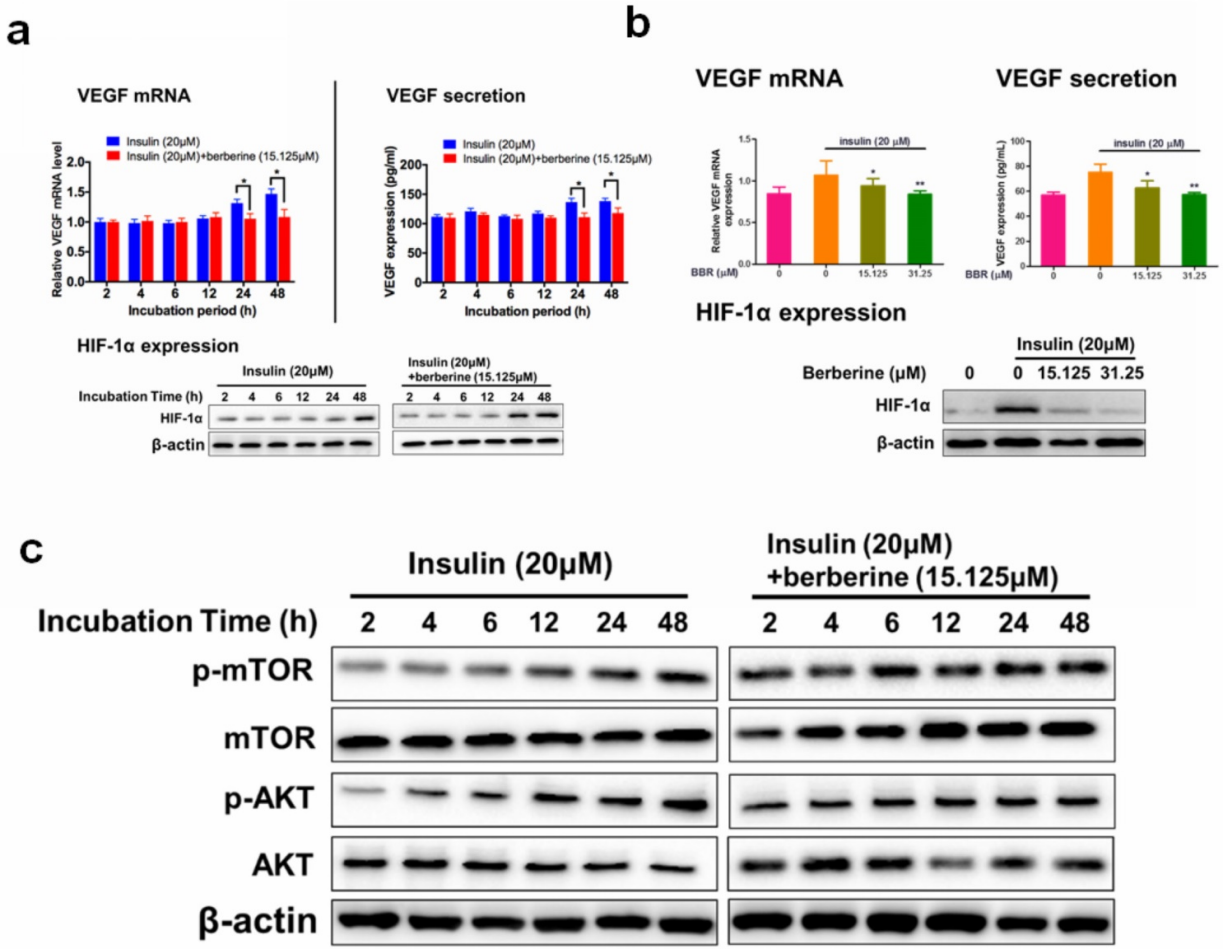
** Berberine suppressed expression of VEGF/ HIF-1α and activity of Akt/mTOR in retina endothelial cells.** Berberine time- **(a)** and dose- **(b)** dependently reduces the expression of VEGF/ HIF-1α in HRECs. **c.** Berberine suppresses the insulin-treated Akt/mTOR pathway.

A few previous studies have shown that BBR inhibited the expression of VEGF and HIF-1α in various cancer cells 30-32. To examine if BBR has any impact on insulin-induced diabetic retinopathy (DR), cytotoxicity of BBR on HRECs cells were measured by the MTT assay. At a dose lower than 31.25 μM, we did not observe any cytotoxicity in HRECs cells (Fig. 2a). The time course of the inhibitory effect of BBR on VEGF/HIF1α expression was examined. We observed that 24 hours and 48 hours of BBR treatment could significantly reduce the expression of VEGF/HIF-1α in HRECs cells (Fig. 2b). At 48 hours of treatment, we observed that BBR at the doses of 15.125 and 31.25 μM or above could significantly inhibit VEGF/HIF-1α expression (Fig. 2c). These observations suggested that BBR was a potent inhibitor of VEGF and HIF-1α in insulin-treated retina endothelial cells.

### The inhibitory effect of BBR on VEGF expression was dependent on Akt/mTOR signalling

**Figure 3:**
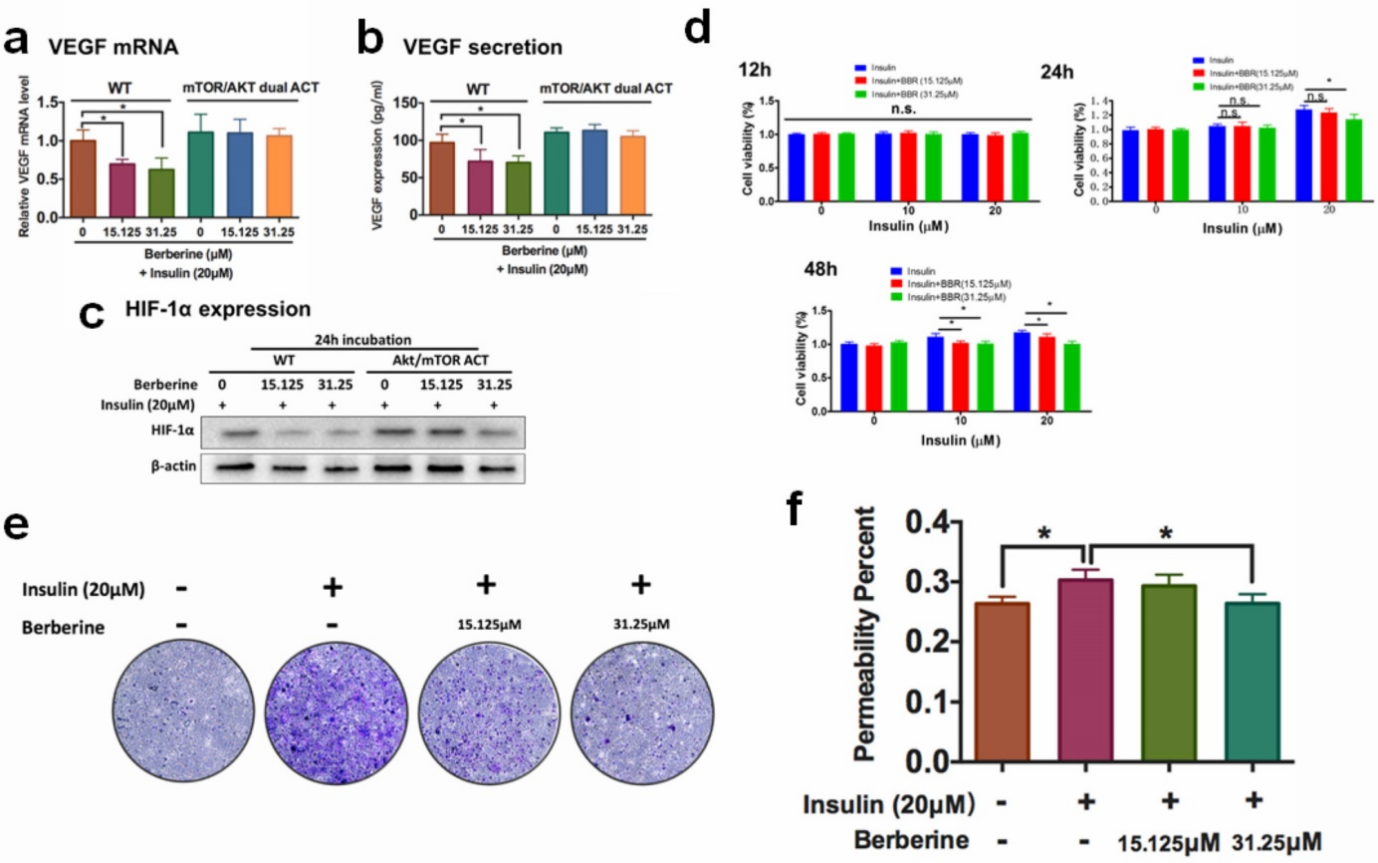
** Berberine suppresses endothelial activation that leads to neovascularization.** Activation of Akt/mTOR pathways attenuated Berberine-induced inhibition of mRNA expression** (a)** and protein expression **(b)** of VEGF, and HIF-1α expression** (c)**. Berberine suppresses the insulin-induced** (d)** proliferation, **(e)** migration, **(f)** permeability of retina endothelial cells.

A previous study revealed that insulin-induced VEGF expression is associated with the activation of AKT/mTOR pathway 13. We examined if the inhibition of BBR on HIF1α/VEGF involves AKT/mTOR. 48 hours of treatment of BBR at the doses of 15.125 μM could significantly suppress insulin-induced the activation of Akt/mTOR pathway (Fig. 3a). It was then concluded that non-toxic doses of BBR significantly suppressed VEGF/HIF1α expression and Akt/mTOR pathway activation in insulin-treated HRECs cells. To further confirm if Akt/mTOR inhibition involves in the inhibitory effect of BBR on VEGF/ HIF1α expression in HREC cells, we then co-expressed constantly activated mutants of Akt and mTOR plasmid (PKB-Myr and mTORC, #86578 and #69015 from Addgene) in HRECs to counteract the effect of BBR, and detected VEGF/HIF1α expression. It was shown that the effect of BBR on VEGF/ HIF1α expression could be significantly abolished by re-activation of Akt/mTOR activity in HRECs cells (Fig. 3b). This suggests that the effect of BBR on VEGF/HIF1α expression was highly dependent on Akt/mTOR pathway.

### BBR suppresses *in vitro* neovascularisation of retina endothelial cells induced by insulin

The activities of proliferation, migration and increased membrane permeability of the retinal endothelial cells are critically involved in retina neovascularization. Activation of the proliferation, migration and increased membrane permeability of the retinal endothelial cells are critically involved in retina neovascularization. We then examined if BBR could suppress the properties of HRECs. The proliferation of HRECs in the presence of insulin alone or co-treatment with BBR was examined by MTT assay; migration of HRECs was measured by transwell assay, and the permeability of the HREC monolayer was examined by the leakage of dextran-FITC. It was shown that at 24 hours and 48 hours, BBR significantly inhibited the proliferation of HREC cells induced by insulin (Fig. 3d); and the presence of BBR could potently block the motility of HREC cells induced by insulin through transwell assay (Fig. 3e). Insulin significantly deteriorated the integrity of the HREC monolayer, and BBR could remarkably recover the monolayer and reduce the leakage of dextran-FITC (Fig. 3f). All these data suggested that the *in vitro* retina neovascularization induced by insulin could be attenuated by BBR treatment.

### BBR suppressed the progression of DR in experimental type I diabetic mice receiving insulin therapy

**Figure 4:**
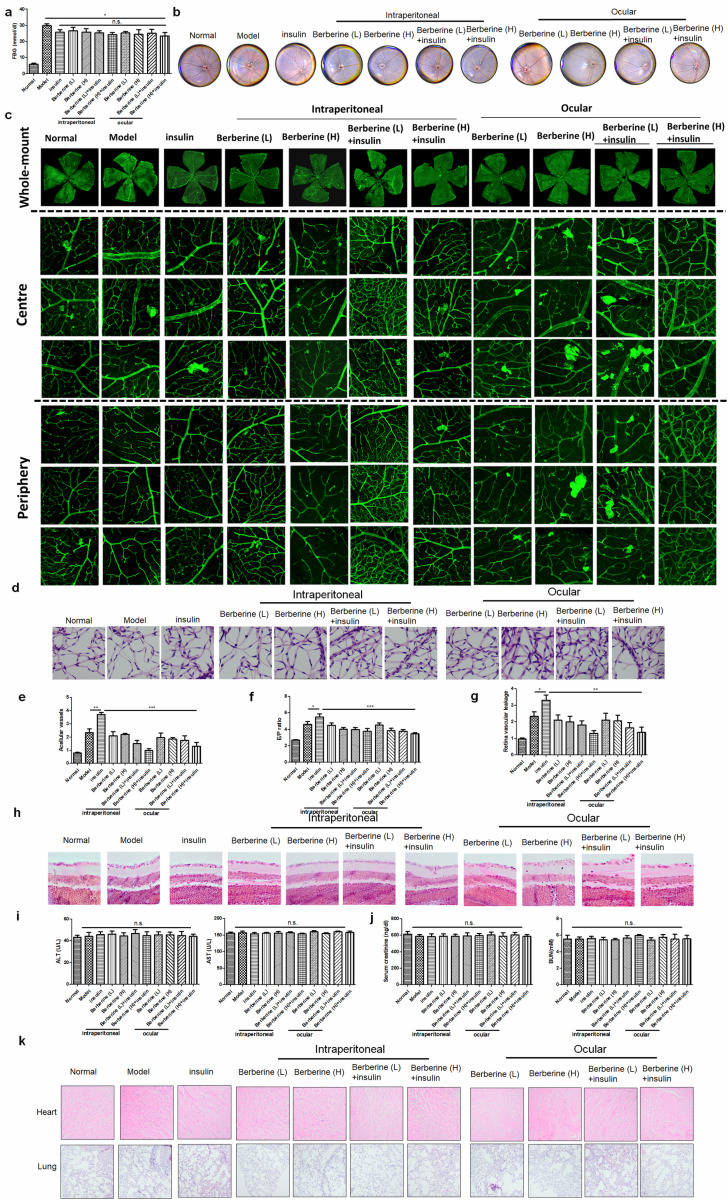
** Berberine suppressed the progression of DR in experimental type I diabetic mice receiving insulin therapy.** Berberine had no further improvement on the glucose control of insulin** (a)** but can improve the fundus condition** (b)** and neovasculature **(c)**. The retina vessel network was prepared** (d)**, which suggested that berberine inhibited acellular capillaries **(e)** or endothelial-to-pericyte ratio **(f)**. Berberine improved retina integrity of insulin-treated mice (g) and survived retina ganglion cells **(h)**. No significant changes of hepatic **(i)** or renal **(j)** index, and histology of major organs **(k)** upon berberine treatment were observed.

Insulin is the first-line treatment for type I diabetes. To examine if BBR can improve the DR in type I diabetic patients receiving insulin therapy, we established STZ-induced experimental type I diabetic model. Treatment of insulin can significantly suppress fasting blood glucose (FBG) in mice, while combination treatment of BBR through oral administration or ocular delivery had minimal improvement on FBG (Fig. 4a). Fundus photography showed that compared with the model group, insulin treatment can increase the numbers of “lipid-laden-like” lesions on the retina, which could be reduced by oral and ocular treatment of BBR (Fig. 4b). The whole-mount of the retina showed much fewer neovascular sprouts in the retina of mice co-treated with insulin and BBR (Fig. 4c). Retinal vasculature was prepared and imaged (Fig. 4d), which revealed that both oral and ocular treatment of BBR can significantly normalize the endothelial dysfunction in mice with DR, including suppression of acellular capillaries and reducing the endothelial cell-to-pericyte (Fig. 4e&4f). BBR treatment could significantly reduce the Evans blue leakage through the retina, suggesting its integrity was protected by BBR treatment (Fig. 4g).

In addition, measurement on the retinal neuron degradation showed that BBR treatment can significantly improve the integrity of the retina ganglion layer of DR mice, as evidenced by the increased number of ganglion cells in the retina of BBR-treated diabetic mice (Fig. 4h). Safety analysis showed that co-treatment of BBR with insulin has no significant effect on tissue histology and blood chemistry of the mice (Fig. 4i-k). These observations revealed that BBR suppressed the progression of DR in the experimental type I diabetic mice receiving insulin therapy.

### BBR suppressed the progression of DR in experimental type II diabetic mice receiving insulin therapy

**Figure 5:**
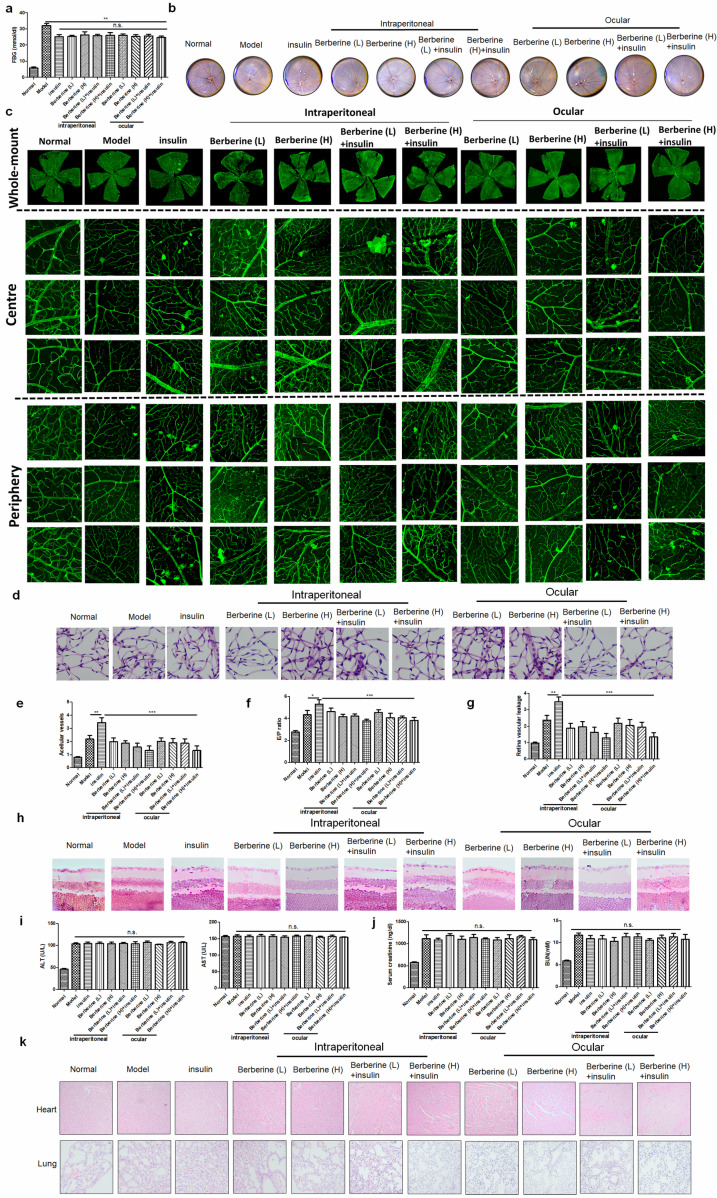
Berberine suppressed the progression of diabetic retinopathy in experimental type II diabetic mice receiving insulin therapy. Berberine had no further improvement on the glucose control of insulin (a) but can improve the fundus condition (b) and neovasculature (c). The retina vessel network was prepared (d), which suggested that berberine inhibited acellular capillaries (e) or endothelial-to-pericyte ratio (f). Berberine improved retina integrity of insulin-treated mice (g) and survived retina ganglion cells (h). No significant changes of hepatic (i) or renal (j) index, and histology of major organs (k) upon berberine treatment were observed.

To extend our observation onto type II diabetes, we established db/db mice receiving insulin treatment. Treatment of insulin can significantly suppress fasting blood glucose (FBG) in mice, while combination treatment of BBR through oral administration or ocular delivery had minimal improvement on FBG (Fig. 5a). Fundus photography showed that compared with the model group, insulin treatment can increase the numbers of “lipid-laden-like” lesions on the retina, which could be reduced by oral and ocular treatment of BBR (Fig. 5b). The whole-mount of the retina showed much fewer neovascular sprouts in the retina of mice co-treated with insulin and BBR (Fig. 5c). Retinal vasculature was prepared and imaged (Fig. 5d), which suggested that both oral and ocular treatment of BBR can improve the acellular capillaries and the endothelial cell-to-pericyte ratio (Fig. 5e&5f), suggesting the improvement of endothelial dysfunction in the retina of type II diabetic mice. BBR treatment could significantly reduce the Evans blue leakage through the retina, suggesting its integrity was protected by BBR treatment (Fig. 5g). BBR treatment by ocular and oral could remarkably maintain the number of ganglion cells in the retina of type II diabetic mice, confirming the improvement of DR by BBR (Fig. 5h). Safety analysis showed that co-treatment of BBR with insulin has no significant effect on tissue histology and blood chemistry of the mice (Fig. 5i-k). These observations revealed that BBR suppressed the progression of DR in the experimental type II diabetic mice receiving insulin therapy.

## Discussion

Glycaemia control remains to be the major approach in the prevention of diabetic complications in both types I and type II diabetes. Various treatments have been developed to suppress blood glucose in diabetic patients, in which insulin is still to be the very effective one 6. However, plenty of clinical evidence has shown that insulin treatment has no beneficial effect on diabetic retinopathy, one of the major complications in diabetic patients; instead, it may even increase the risk of incidence of this disease 7. This contradicting activity of insulin is yet to be scientifically understood due to the complex structure of the retina with multiple types of cells involved in the pathogenic development of diabetic retinopathy. In our study, we systematically investigated the effect of insulin on different types of retina cells, in which we found that retina endothelial cells are uniquely activated upon insulin exposure. Retina endothelial cells are a type of cells involved in both non-progressive and progressive diabetic retinopathy via initiating retina vasculature. The proliferation, migration and permeability of endothelial retina cells facilitate breakdown retina barrier, recruiting more pro-inflammatory immune cells and cytokines into the retina, and therefore worsening the disease progression 33. Our finding that insulin can directly activate Akt/mTOR signalling and induce expression of HIF-1α/VEGF *in vivo* retina endothelial cells, echoes our *in vivo* observation that long-term treatment of insulin may increase the risk of neovascularization in the retina of diabetic animals, and provide scientific evidence to support the claims of the unfavourable effect of insulin in diabetic retinopathy control.

**Figure 6:**
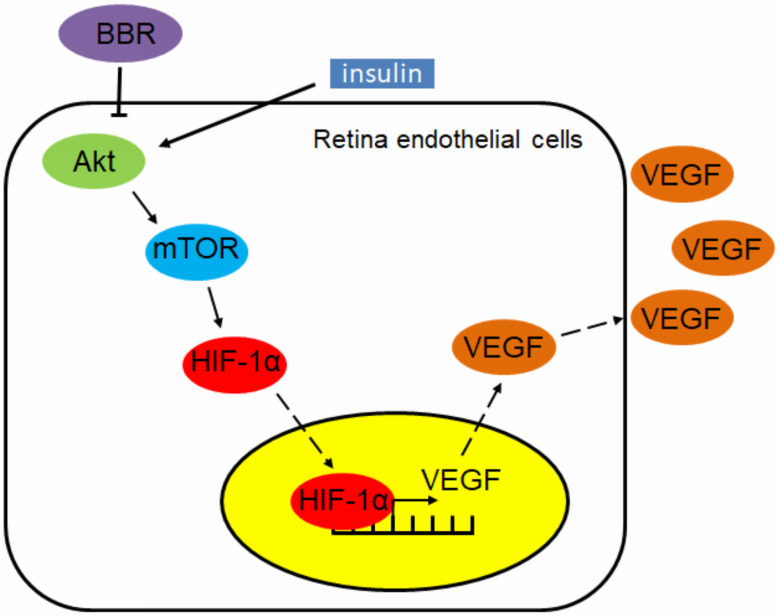
Schematic mechanism underlying the regulatory effect of berberine on insulin-induced diabetic retinopathy

Our finding also suggests that berberine can effectively control the progression of diabetic retinopathy in insulin-treated diabetic mice. This effect can be interpreted by the *in vitro* observation that berberine suppresses insulin-induced activation of retina endothelial cells through Akt/mTOR/HIF-1α/VEGF pathways (Fig. 6). However, our study does not exclude any other possible action of berberine that may contribute to its beneficial effects on diabetic retinopathy. As a natural alkaloid and over-the-counter drug used for the treatment of gastrointestinal diseases in China, berberine was well documents for its anti-inflammatory activity.

Suppression of inflammation by berberine has been proven previously in different types of diseases that contribute to its improvement of health conditions 34. In our study, although we did not examine if the anti-inflammatory effect of berberine can relieve retina conditions in diabetic animals, it would not be surprised retina inflammation can be suppressed by its treatment. Retina inflammation is another important process that mediates the progression of diabetic retinopathy, and indeed it is yet conclusive whether retina inflammation or neovascularization is the predominant factor in the disease progression.

Crosstalk between the two pathogenic signs of progress has been extensively discussed 35. In the case of insulin-associated progression of diabetic retinopathy, it is more likely that retina endothelial activation plays a leading role, as our *in vitro* observation suggests no involvement of microglia activation. However, this did not reject the possibility *in vivo* that berberine can suppress inflammation and as a result substantially contribute to the pharmacological action. The possible action of berberine in suppressing retina inflammation may deserve an individual investigation in the future and successful and systematic demonstration of the action mechanism of berberine may facilitate its development as a complementary therapy of insulin in improving complications like diabetic retinopathy.

Taken together, in this study we reported the novel function of BBR in improving insulin therapy-induced diabetic retinopathy in both type I and II DM animals. Experimentally, we proved that insulin treatment can induce retina endothelial cell activation, which was related to the induced expression of HIF-1α and VEGF. Berberine treatment and dose- and time-dependently suppressed insulin-induced expression of HIF-1α and VEGF in retina endothelial cells. Berberine suppressed Akt/mTOR signal activity, and constitutive activation of Akt/mTOR signalling abolished berberine-induced inhibition of HIF-1α and VEGF in retina endothelial cells. BBR can improve diabetic retinopathy in type I and II diabetic animals receiving insulin therapy, reducing neovasculature and neural cell damage in the eyes and therefore supplemented the therapeutic outcome of insulin therapy.
